# Supplementary material for: A clinical deep learning framework for continually learning from cardiac signals across diseases, time, modalities, and institutions
Source: Nat Commun. 2021 Jul 9;12:4221. doi: 10.1038/s41467-021-24483-0 (PMC8270996; doi:10.1038/s41467-021-24483-0)
Supplement: Supplementary file 1 — Supplementary Information [file 41467_2021_24483_MOESM1_ESM.pdf]

Supplementary Information - A Clinical Deep Learning Framework for Continually Learning from Cardiac Signals Across Diseases, Time, Modalities, and Institutions

Dani Kiyasseh[1,*], Tingting Zhu[1], and David Clifton[1]

[1]University of Oxford, Department of Engineering Science, Oxford, OX3 7DQ, United Kingdom
[*]dani.kiyasseh@eng.ox.ac.uk

Supplementary Note 1 - Methods

The two ideas underlying our proposal are the storage of instances into *and* the acquisition of instances from a buffer such that destructive interference is mitigated. We summarize these in the algorithms shown below.

Algorithms

Algorithm 1 CLOPS

Input: MC epochs τ_{MC}, sample epochs τ_S, MC samples T, storage fraction b, acquisition fraction a, task data $\mathscr{D}_T$, buffer $\mathscr{D}_B$, training epochs per task τ

```
for (x,y) ~ D_T do
    calculate L using eq. 5
    update β_ij                                        ▷ update task-instance parameters
    if epoch = τ then
        D_B = StoreInBuffer(D_B, β_iT, b, D_T)
    if epoch in τ_MC then
        G = MonteCarloSamples(D_B)
    if epoch in τ_S then
        D_T = AcquireFromBuffer(D_B, G, a, D_T)
```

Algorithm 2 StoreInBuffer

Input: buffer $\mathscr{D}_B$, task-instance parameters $\beta_{i\mathscr{T}}$, storage fraction b, task data $\mathscr{D}_T$

```
calculate s_iT using eq. 6
SortDescending(s_iT)
(x_b, y_b) ⊂ D_T
D_B ∈ (D_B ∪ (x_b, y_b))
```

Algorithm 3 MonteCarloSamples

Input: buffer $\mathscr{D}_B$

```
for (x,y) ~ D_B do
    for MC sample in T do
        obtain p(y|x, ω̂) and store in G ∈ R^{MxTxC}
```

Algorithm 4 AcquireFromBuffer

Input: buffer $\mathscr{D}_B$, MC posterior distributions G, acquisition fraction a, task data $\mathscr{D}_T$
1: calculate α using eq. 7
2: SortDescending(α)
3: $(x_a, y_a) \subset \mathscr{D}_B$
4: $\mathscr{D}_T \in (\mathscr{D}_T \cup (x_a, y_a))$

Supplementary Note 2 - Datasets

Data Preprocessing

In this section, we describe in detail each of the datasets used in our experiments in addition to any pre-processing that is applied to them.

Cardiology ECG, $\mathscr{D}_1$[1]. Each ECG recording was originally 30 seconds with a sampling rate of 200Hz. Each ECG frame in our setup consisted of 256 samples resampled to 2500 samples. Labels made by a group of physicians were used to assign classes to each ECG frame depending on whether that label coincided in time with the ECG frame. These labels are: AFIB, AVB, BIGEMINY, EAR, IVR, JUNCTIONAL, NOISE, NSR, SVT, TRIGEMINY, VT, and WENCKEBACH. Sudden bradycardia cases were excluded from the data as they were not included in the original formulation by the authors. The ECG frames were not normalized.

Chapman ECG, $\mathscr{D}_2$[2]. Each ECG recording was originally 10 seconds with a sampling rate of 500Hz. We downsample the recording to 250Hz and therefore each ECG frame in our setup consisted of 2500 samples. We follow the labelling setup suggested by[2] which resulted in four classes: Atrial Fibrillation, GSVT, Sudden Bradychardia, Sinus Rhythm. The ECG frames were not normalized.

PhysioNet 2020 ECG, $\mathscr{D}_3$[3]. Each ECG recording varied in duration from 6 seconds to 60 seconds with a sampling rate of 500Hz. Each ECG frame in our setup consisted of 2500 samples (5 seconds). We assign multiple labels to each ECG recording as provided by the original authors. These labels are: AF, I-AVB, LBBB, Normal, PAC, PVC, RBBB, STD, and STE. The ECG frames were normalized in amplitude between the values of 0 and 1.

PhysioNet 2017 ECG, $\mathscr{D}_4$[4]. Each ECG recording varies in length between 9 and 30 seconds with a sampling rate of 300Hz. Each ECG frames in our setup consists of 2500 samples. We use the original labels, resulting in four classes: Normal, AF, Other, and Noisy. The ECG frames are not normalized.

Dataset Splis

In this section, we outline in Table 4 the number of instances present in each of the training, validation, and test sets of the various datasets. Data were split into these sets according to a 60, 20, 20 configuration and were always performed at the patient-level. In other words, a patient did not appear in more than one set.

Supplementary Table 1. Number of instances (number of patients) in the training, validation, and test splits for datasets $\mathscr{D}_1$ to $\mathscr{D}_3$. The number of instances for $\mathscr{D}_3$ are shown for one lead.

Dataset	Train	Validation	Test
$\mathscr{D}_1$	4079 (180)	1131 (50)	1386 (62)
$\mathscr{D}_2$	76,606 (6387)	25,535 (2129)	25,555 (2130)
$\mathscr{D}_3$	11,598 (4402)	3238 (1100)	4041 (1375)
$\mathscr{D}_4$	16,365	4,582	5,824

Task Splits

We outlined three continual learning scenarios, each of which comprise sequential tasks. In turn, each of these tasks consist of their own training, validation, and test sets that are leveraged throughout the evaluation process. In Table 2, we present the number of instances available in these sets.

Supplementary Table 2. Number of instances in the training, validation, and test sets of tasks in the three continual learning scenarios; Class-IL, Time-IL, Domain-IL, and Institute-IL.

Task Name	0-1	2-3	4-5	6-7	8-9	10-11
Training	781	227	463	2118	309	179
Validation	126	141	118	587	83	82
Test	285	77	130	703	89	102

(a) Class-IL

Task Name	Term 1	Term 2	Term 3
Training	37596	12534	12552
Validation	20586	6858	6864
Test	18424	6143	6139

(b) Time-IL

Task Name	Lead I	Lead II	Lead III	...	Lead V6
Training	11598	11598	11598	...	11598
Validation	3238	3238	3238	...	3238
Test	4041	4041	4041	...	4041

(c) Domain-IL

Task Name	Chapman	PhysioNet 2017	Cardiology
Training	37596	16365	4079
Validation	20586	4582	1131
Test	18424	5824	1386

(d) Institute-IL

Supplementary Note 3 - Implementation Details

In this section, we outline the architecture of the neural network used for all experiments. We chose this architecture due to its simplicity and its simultaneous ability to learn on the datasets provided. We also outline the batchsize and the learning rate used for training on the various datasets.

Network Architecture

Supplementary Table 3. Network architecture used for all experiments. K, C_{in}, and C_{out} represent the kernel size, number of input channels, and number of output channels, respectively. A stride of 3 was used for all convolutional layers.

Layer Number	Layer Components	Kernel Dimension
1	Conv 1D<br>BatchNorm<br>ReLU<br>MaxPool(2)<br>Dropout(0.1)	7 x 1 x 4 (K x C_{in} x C_{out})
2	Conv 1D<br>BatchNorm<br>ReLU<br>MaxPool(2)<br>Dropout(0.1)	7 x 4 x 16
3	Conv 1D<br>BatchNorm<br>ReLU<br>MaxPool(2)<br>Dropout(0.1)	7 x 16 x 32
4	Linear<br>ReLU	320 x 100
5	Linear	100 x C (classes)

Experiment Details

Supplementary Table 4. Batchsize and learning rates used for training with different datasets. The Adam optimizer was used for all experiments.

Dataset	Batchsize	Learning Rate
$\mathscr{D}_1$	16	10^{-4}
$\mathscr{D}_2$	256	10^{-4}
$\mathscr{D}_3$	256	10^{-4}
$\mathscr{D}_4$	256	10^{-4}

Baseline Replay-based Methods

In this section, we outline how we have adapted two replay-based methods for application in our continual learning scenarios. This adaptation was necessary since both of these methods were designed for a more extreme online setting whereby instances are only seen once and never again.

GEM. At the end training on each task, we randomly select a subset of the instances and store them in the buffer. Since our data are shuffled, this is equivalent to the strategy proposed by the original authors which involves storing the most recent instances. On subsequent tasks, and at each iteration, we check the gradient dot product requirement outlined by the original authors, and if that is violated, we solve a quadratic programming problem. This implementation is exactly the same as that found in the original MIR manuscript.

MIR. At the end of training on each, we randomly select a subset of the instances and store them in the buffer. This is more suitable for our scenarios relative to reservoir sampling, which was implemented by the original authors. As our approach is task-aware, our buffer is task-aware, unlike the original MIR implementation. Such information would only strengthen the performance of our adapted version. On subsequent tasks, and at each iteration, we randomly select a subset of the instances from the buffer, and score them according to the metric proposed by the original authors. We sort these instances in descending order of the scores and acquire the top scoring instances from each task. We ensure that the total number of instances replayed is equal to the number of instances in the mini-batch of the current task.

Evaluation Metrics

Metrics used to evaluate continual learning methodologies are of utmost importance as they provide us with a glimpse of the strengths and weaknesses of various learning strategies. In this section, we outline the traditional metrics used within continual learning. We argue that such metrics are limited in that they only capture the global behaviour of strategies. Consequently, we propose two more fine-grained metrics below, BWT_t and BWT_λ.

Average AUC. Let R_j^i represent the performance of the network in terms of AUC on task j after having been trained on task i.

$$\text{Average AUC} = \frac{1}{N}\sum_{j=1}^{N}\text{R}_j^N \tag{1}$$

Backward Transfer. $\text{BWT} \in [-1, 1]$ is used to quantify the degree to which training on subsequent tasks affects the performance on previously-seen tasks. Whereas positive BWT is indicative of constructive interference, negative BWT is indicative of destructive interference.

$$\text{BWT} = \frac{1}{N-1}\sum_{j=1}^{N-1}\text{R}_j^N - \text{R}_j^j \tag{2}$$

t-Step Backward Weight Transfer. To determine how performance changes "t-steps into the future", we propose BWT_t which evaluates the performance of the network on a previously-seen task, after having trained on t-tasks after it.

$$\text{BWT}_t = \frac{1}{N-t}\sum_{j=1}^{N-t}\text{R}_j^{j+t} - \text{R}_j^j \tag{3}$$

Lambda Backward Weight Transfer. We extend BWT_t to all time-steps, t, to generate BWT_λ. As a result, we can identify improvements in methodology at the task-level.

$$\text{BWT}_\lambda = \frac{1}{N-1}\sum_{j=1}^{N-1}\left[\frac{1}{N-j}\sum_{t=1}^{N-j}\text{R}_j^{j+t} - \text{R}_j^j\right] \tag{4}$$

Supplementary Note 4 - Results

In the main manuscript, we conduct experiments in three continual learning scenarios: 1) Class-IL, 2) Time-IL, and 3) Domain-IL. For each, we illustrate the learning curves during training and the corresponding evaluation metric values. In this section, we present results that are complementary to those found in the main manuscript. More specifically, we compare the performance of CLOPS to that of the baseline methods implemented.

Time-IL

In this section, we illustrate the performance of the baseline MIR system in the Time-IL scenario.

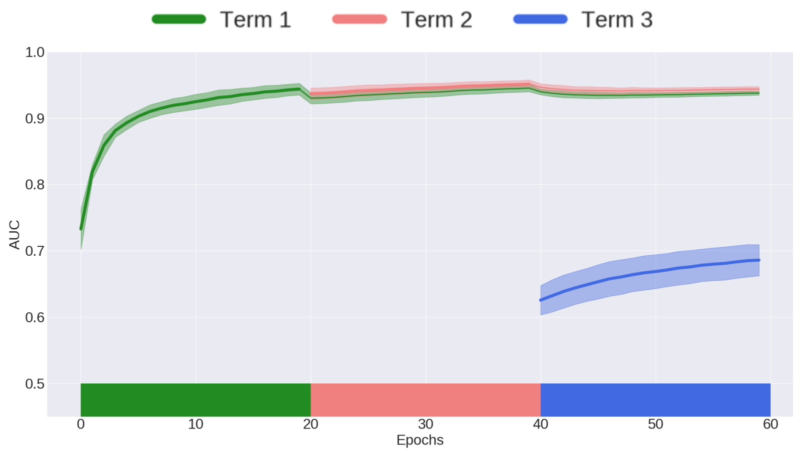

Supplementary Figure 1. Mean validation AUC of MIR in the Time-IL scenario. Results are shown as a function of storage fractions, *b*, and acquisition fractions, *a* and are an average across five seeds. The shaded area represents one standard deviation from the mean.

Domain-IL

To gain a better understanding of the learning dynamics of CLOPS in the domain-IL scenario, we plot the validation AUC in Fig. 2. Here, significant destructive interference occurs in the fine-tuning strategy. This is shown by large drops in the AUC of one task when subsequent tasks are trained on. For instance, when training on Lead V1 (starting at epoch 240), performance on Lead I (green) drops from an AUC $= 0.725 \rightarrow 0.475$, completely erasing any progress that had been made on that lead. We hypothesize that this is due to the different representations of instances belonging to Lead V1 and Lead I. Anatomically speaking, these two leads are projections of the same electrical signal in the heart that are oriented approximately 180 degrees to one another. This behaviour is absent for almost all leads when CLOPS is implemented, as shown in Fig. 2b. A notable exception is Lead aVR at epoch 200 where performance drops from an AUC $\approx 0.75 \rightarrow 0.65$.

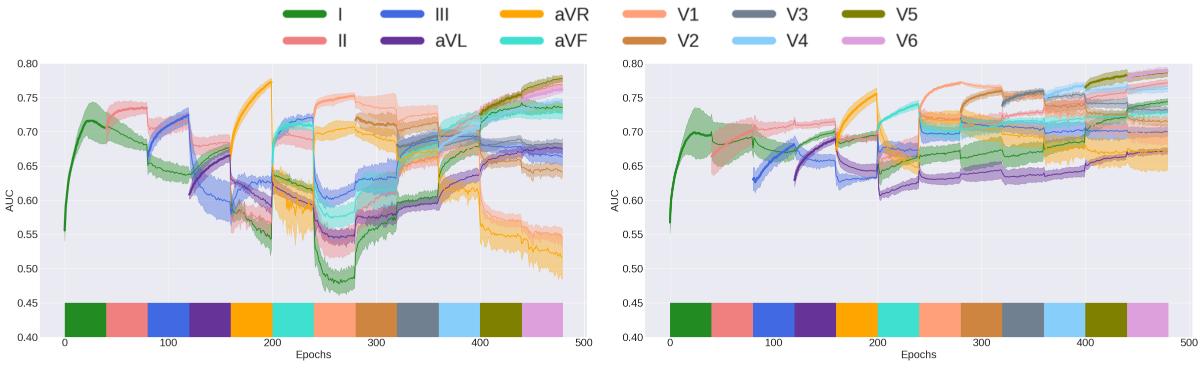

(a) Fine-tuning **(b)** CLOPS

Supplementary Figure 2. Mean validation AUC of a) fine-tuning and b) CLOPS ($b = 0.25$ and $a = 0.50$) strategy in the Domain-IL scenario. Each task belongs to the same dataset yet different input modality. Coloured blocks indicate tasks on which the learner is currently being trained. The shaded area represents one standard deviation from the mean across 5 seeds.

Effect of Task Order

The order in which tasks are presented to a continual learner can significantly impact the degree of destructive interference. To quantify this phenomenon and evaluate the robustness of CLOPS to task order, we repeat the Class-IL experiment conducted in the main manuscript after having randomly shuffled the tasks.

Class IL

Task order does have an effect on destructive interference. By comparing the evaluation metric values shown in Table 5 with the results in the main manuscript, we show that poorer generalization performance is achieved when learning sequentially on tasks that have been randomly shuffled. Given that one has limited control over the order in which data is streamed, continual learning approaches must be robust to task order. Indeed, we claim that CLOPS is robust to such changes. This can be seen by its achievement of an AUC $= 0.796$ regardless of task order (in Table 1 in main manuscript and Table 5).

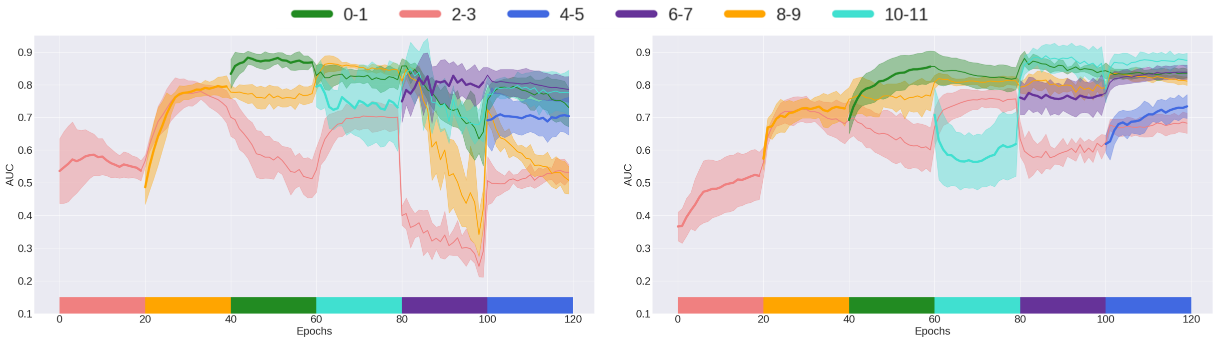

(a) Fine-tuning **(b)** CLOPS

Supplementary Figure 3. Mean validation AUC of a) fine-tuning strategy and b) CLOPS ($b = 0.25$ and $a = 0.50$) in the Class-IL scenario with 6 tasks after being *randomly re-ordered*. Each task belongs to a mutually-exclusive pair of classes from $\mathscr{D}_1$. Coloured blocks indicate tasks on which the learner is currently being trained. The shaded area represents one standard deviation from the mean across 5 seeds.

Supplementary Table 5. Performance of CL strategies in the Class-IL scenario. Storage and acquisition fractions are $b = 0.25$ and $a = 0.50$, respectively. Results are shown across five seeds.

Method	Average AUC	BWT	BWT_t	BWT_λ
Fine-tuning	0.672 ± 0.022	-0.081 ± 0.049	0.014 ± 0.034	-0.055 ± 0.025
CLOPS	**0.796 ± 0.006**	**0.110 ± 0.021**	**0.096 ± 0.025**	**0.095 ± 0.036**

Distribution of Task-Instance Parameters. We also illustrate the distribution of the tracked task-instance parameters in Fig. 4. These distributions differ in terms of overlap compared to those in Fig. 4 of the main manuscript. They both, however, follow a Gaussian distribution and roughly maintain their relative locations to one another. For instance, task $[6,7]$ and $[10,11]$ consistently generate the lowest and highest s values, respectively, regardless of task order. Such a finding illustrates that task-instance parameters are agnostic to task-order, which is a desirable trait when attempting to quantify task difficulty.

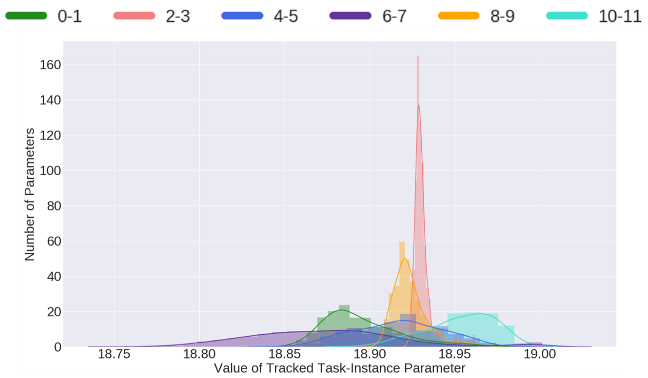

Supplementary Figure 4. Distribution of the tracked task-instance parameter values, s, corresponding to our proposed strategy in the Class-IL scenario with 6 tasks. Storage and acquisition fractions are $b = 0.25$ and $a = 0.50$, respectively. Each colour corresponds to a different task. Results are shown for one seed.

Qualitative Evaluation of Task-Instance Parameters, β

In this section, we aim to qualitatively evaluate the interpretation of task-instance parameters as proxies for task-difficulty. To do so for the various CL scenarios, we plot two ECG tracings that correspond to the highest and lowest s values (see eq. 6). As instances with low s values should be more difficult to classify, these ECG tracings might be expected to exhibit abnormalities that make it difficult for a cardiologist to correctly diagnose. Conversely, ECG tracings with high s values should be easy to classify from an expert's perspective. We illustrate these tracings, which are coloured based on the task to which they belong, for the Time-IL and Domain-IL scenarios.

Time-IL

In Fig. 5, we see that the network had a difficult time classifying the ECG tracing that corresponds to the lowest s value with a ground truth label of GSVT (Supra-ventricular Tachycardia). On the other end of the spectrum, the network was able to comfortably classify the ECG tracing that corresponds to the highest s value with a ground truth label of SB (Sudden Bradycardia).

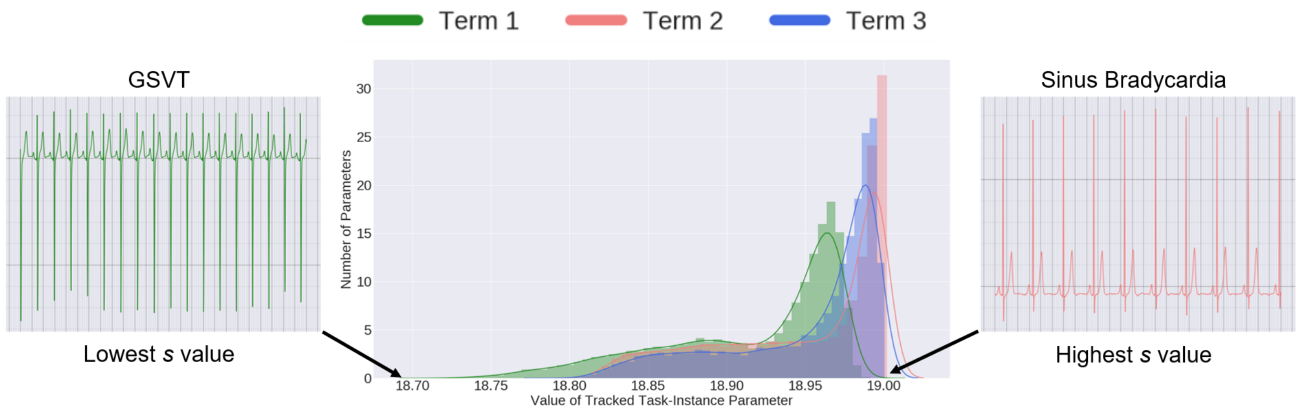

Supplementary Figure 5. Distribution of the tracked task-instance parameter values, s, corresponding to our proposed strategy in the Time-IL scenario. Each colour corresponds to a different task. Results are shown for one seed. The ECG tracings correspond to the lowest and highest s values.

Domain-IL

In Fig. 6, we see that the network had a difficult time classifying the ECG tracing that corresponds to the lowest *s* value with a ground truth label of AF (Atrial Fibrillation). This could be due to the amount of noise present in the tracing. On the other end of the spectrum, the network was able to comfortably classify the ECG tracing that corresponds to the highest *s* value with a ground truth label of AF also.

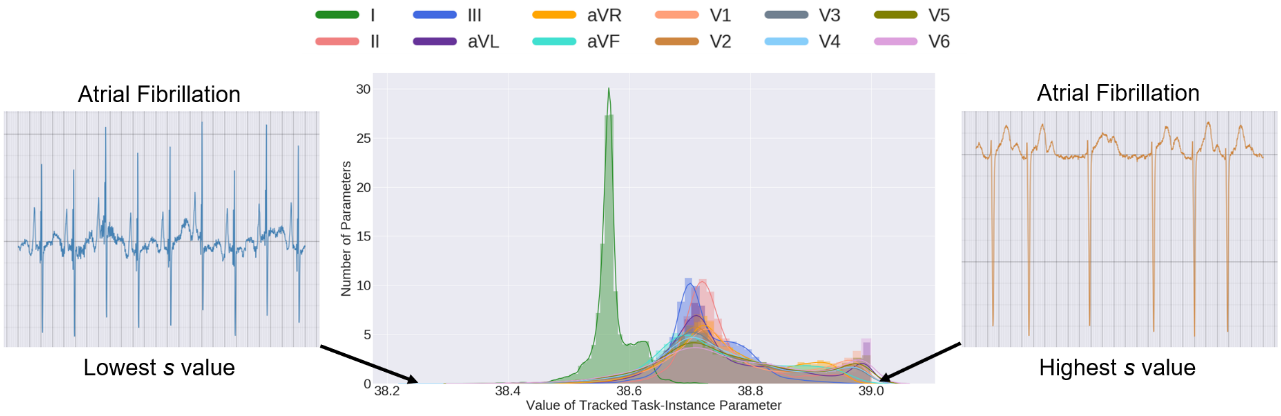

Supplementary Figure 6. Distribution of the tracked task-instance parameter values corresponding to our proposed strategy in the Domain-IL scenario. Each colour corresponds to a different task. Results are shown for one seed. The ECG tracings correspond to the lowest and highest *s* values.

Effect of Storage Function Form

We proposed the following storage function as a way to guide the storage of instances into the replay buffer. This function is dependent upon the task-instance parameters, β, as they have been tracked throughout the training process.

$$s_{i\mathcal{T}} = \int_0^{\tau} \beta_{i\mathcal{T}}(t)dt \approx \sum_{t=0}^{\tau} \left(\frac{\beta_{i\mathcal{T}}(t+\Delta t) + \beta_{i\mathcal{T}}(t)}{2} \right) \Delta t \tag{5}$$

In this section, we aim to quantify the effect of the form of the storage function on the generalization performance of our network. More specifically, we explore a different form of the storage function where the task-instance parameters, β, are squared before the trapezoidal rule is applied. Mathematically, the storage function now takes on the following form.

$$s_{i\mathcal{T}} = \int_0^{\tau} \beta_{i\mathcal{T}}^2(t)dt \approx \sum_{t=0}^{\tau} \left(\frac{\beta_{i\mathcal{T}}^2(t+\Delta t) + \beta_{i\mathcal{T}}^2(t)}{2} \right) \Delta t \tag{6}$$

To compare these two storage functions, we conduct experiments in the Class-IL scenario as we vary the storage fraction, b, and acquisition fraction, a. In Fig. 7, we present the validation AUC of these two experiments conducted across five seeds. We find that our proposed storage function, and the one used throughout the manuscript (Eq. 5), is more advantageous than that shown in Eq. 6. This is evident by the consistently higher Average AUC scores. A possible explanation for this observation is the following. Recall that task-instance parameters, β, are a rough proxy for the difficulty of an instance to be classified. Therefore, when ranking such instances based on Eq. 5 for storage purposes, we are effectively storing the relatively 'easiest-to-classify' instances into the buffer. We hypothesize that upon squaring the task-instance parameters, as is done in Eq. 6, this interpretation no longer holds. As a result, the discrepancy between instances diminishes and thus hinders the storage process.

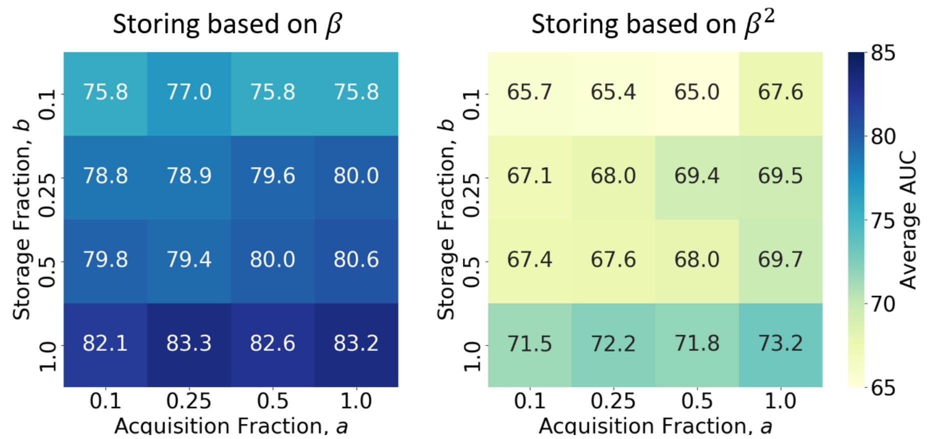

Supplementary Figure 7. Effect of form of storage function on the generalization performance of the network. Mean validation AUC when buffer storage is based on **(Left)** Eq. 5 and **(Right)** Eq. 6. We show that our proposed storage function, and the one used throughout the manuscript (Eq. 5), is more advantageous than that shown in Eq. 6.

Effect of Storage Fraction, b, and Acquisition Fraction, a, on Performance

Replay-based continual learning strategies can be computationally expensive and resource-hungry due to the presence of a buffer and the need to acquire instances from it. To map out the performance of CLOPS under various resource constraints, we set out to investigate the effect of changes in the storage and acquisition fractions on the various evaluation metrics.

To simultaneously validate our decision of storing the top b instances from each task into the buffer, we conduct the aforementioned experiments in two scenarios: 1) The first scenario involves sorting the instances according to their s value (eq. 6) and storing the top b fraction of instances into the buffer (**Storing top *b* fraction**). 2) The second scenario involves storing the bottom b fraction of instances (**Storing bottom *b* fraction**). By conducting this specific experiment, we look to determine the relative benefit of replaying either relatively easy or difficult instances.

Average AUC

Larger storage and acquisition fractions should further alleviate destructive interference and improve generalization performance. The intuition is that large fractions will expose the learner to a more representative distribution of data from previous tasks. We quantify this graded response in Fig. 8 where the AUC $= 0.758 \rightarrow 0.832$ as $b, a = 0.1 \rightarrow 1$. We also claim that a performance bottleneck lies at the storage phase. This can be seen by the larger improvement in performance as a result of an increased storage fraction compared to that observed for a similar increase in acquisition fraction. Despite this, a strategy with fractions as low as $b = 0.25$ and $a = 0.1$ is sufficient to outperform the fine-tuning strategy.

In addition to exploring the graded effect of fraction values, we wanted to explore the effect of storing the bottom, most difficult, b fraction of instances in a buffer. The intuition is that if a learner can perform well on these difficult replayed instances, then strong performance should be expected on the remaining relatively easier instances. We show in Fig. 8 (right) that although the performance seems to be on par with that in Fig. 8 (left) for $b = (0.5, 1)$, the most notable differences arise at fractions $b < 0.5, a < 0.5$ (red box). We believe this is due to the extreme 'hard-to-classify' nature of instances with low s values. These findings justify our storing of the top b fraction of instances.

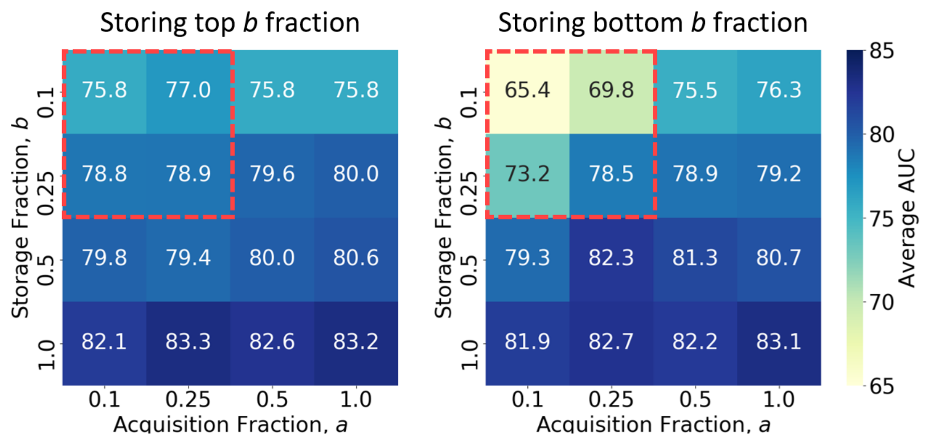

Supplementary Figure 8. Mean validation AUC of CLOPS in the Class-IL scenario implemented while either storing b instances with the highest s value (left) or lowest s value (right). Please refer to eq. 6 for the definition of s. Results are shown as a function of storage fractions, b, and acquisition fractions, a and are an average across five seeds. Darker coloured cells indicate higher generalization performance.

Backward Weight Transfer

In this section, we illustrate the results of the two scenarios 1) Storing top b fraction and 2) Storing bottom b fraction on backward transfer. In the top left heatmap, we show that as the storage fraction $b = 0.1 \rightarrow 1$ at an acquisition fraction, $a = 0.1$, is associated with improved constructive interference (BWT $= 0.034 \rightarrow 0.066$). Although this is also true for the scenario in the second column, we show that performance is worse in this case. Such a finding corroborates our claim in the main manuscript that storing the top instances, which correspond to the "easiest-to-classify" instances, is more beneficial in the context of CL. This provides further evidence that supports our use of the buffer-storage strategy represented by the first column for all experiments conducted in the main manuscript.

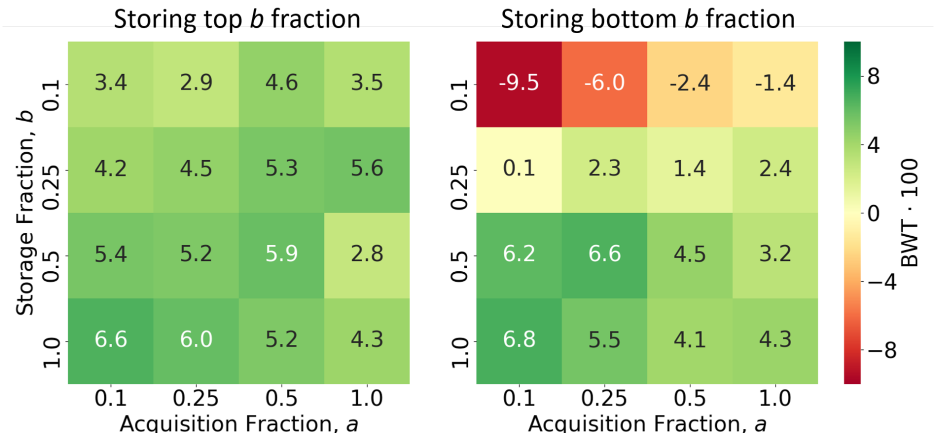

(a) BWT

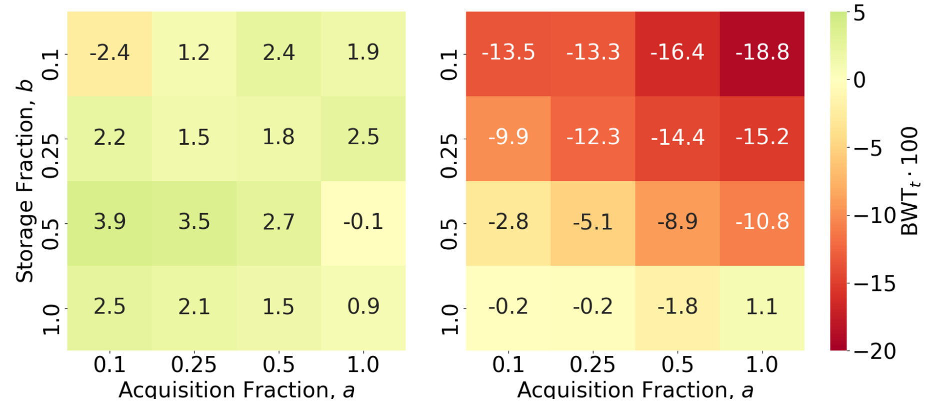

(b) BWT_t

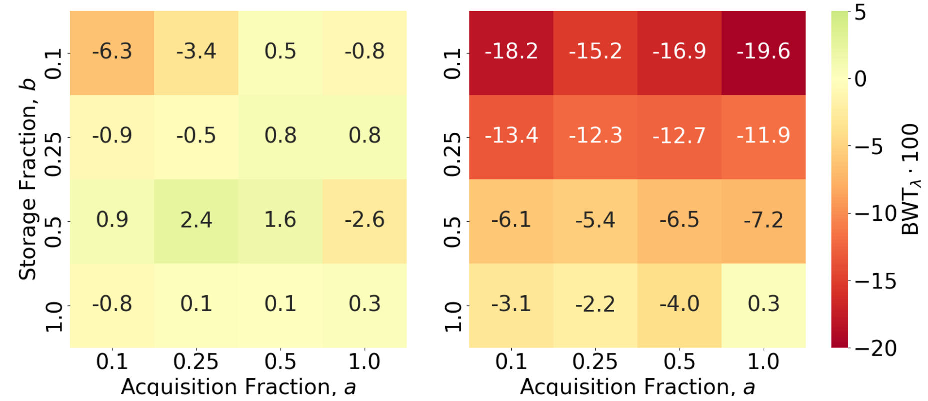

(c) BWT_λ

Supplementary Figure 9. Backward transfer on the validation set using CLOPS while storing top b fraction of instances (left column) and bottom b fraction of instances (right column) in the Class-IL scenario. Results are shown as a function of storage fractions, b, and acquisition fractions, a and are an average across five seeds.

Effect of Task-Instance Parameters, β, and Acquisition Function, α

In this section, we illustrate the effect of two ablation studies on backward transfer. **Random Storage** (RS) is a training procedure that stores instances randomly into a buffer yet acquires them using an acquisition function. **Random Acquisition** (RA), on the other hand, employs task-instance parameters for buffer-storage yet acquires instances randomly from the buffer.

When comparing the BWT_{λ} heatmaps, we show that the random storage scenario leads to greater destructive interference compared to random acquisition. This can be seen at low storage and acquisition fractions ($b < 0.5$ and $a < 0.5$). Since RA and RS can be thought of as "smart" storage and "smart" acquisition, respectively, the superiority of the former implies that a storage strategy is more important than an acquisition strategy in this context and when evaluated from the perspective of backward transfer. With most recent CL replay strategies solely focusing on acquisition, our finding suggests that further research into storage functions could be of value.

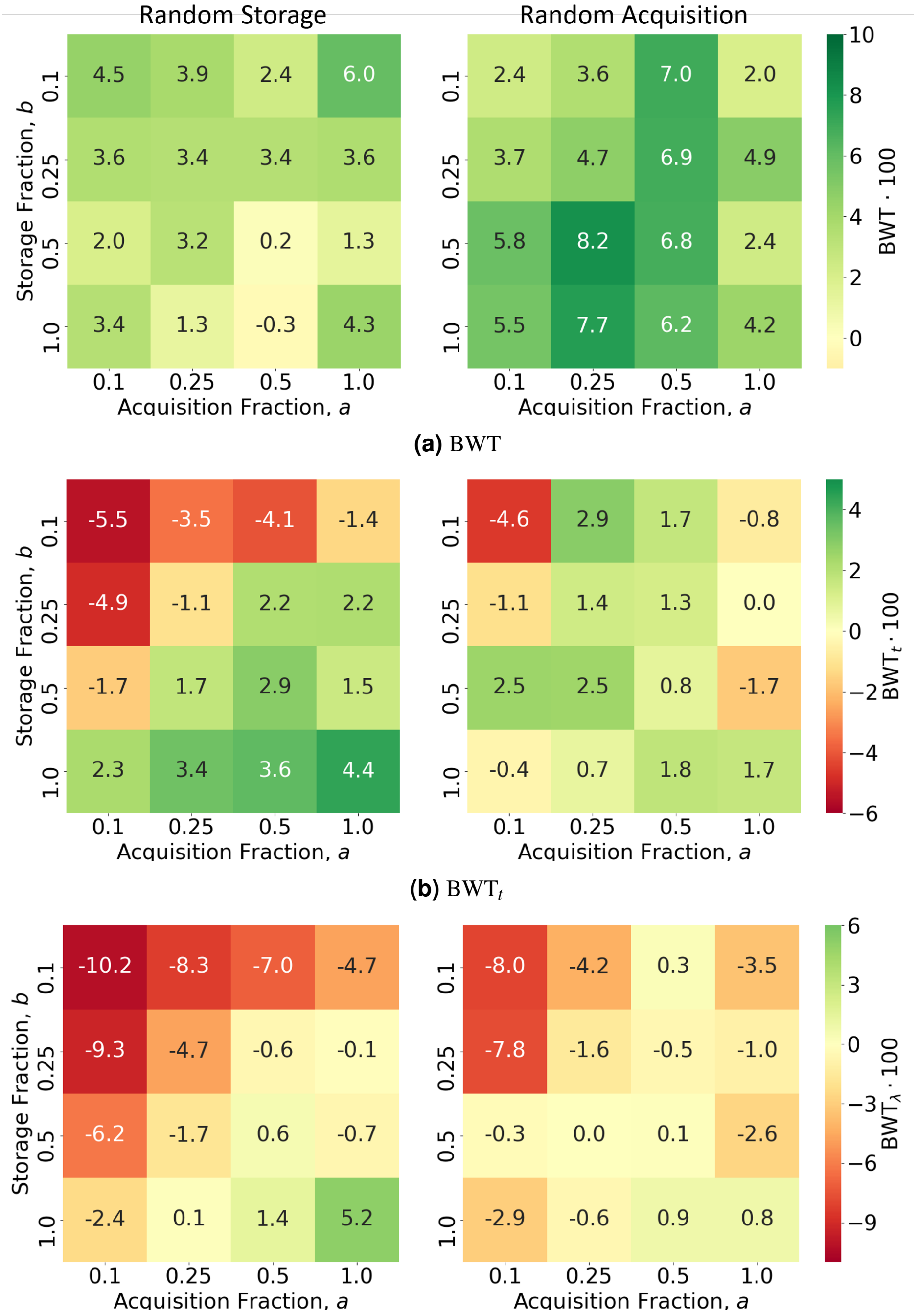

(a) BWT

(b) BWT_t

(c) BWT_λ

Supplementary Figure 10. Backward transfer on the validation set using a Random Storage (left column) and Random Acquisition (right column) strategy in the Class-IL scenario. Results are shown as a function of storage fractions, *b*, and acquisition fractions, *a* and are an average across five seeds.

Effect of Weighting Replayed Instances

In our formulation, we stated that replayed instances are *not* weighted with task-instance parameters. Our hypothesis was that this would negatively interfere with the learning process on subsequent tasks. To quantify the effect of such a weighting, we perform several experiments in which replayed instances are weighted according to their corresponding task-instance parameters. Notably, we freeze these parameters and no longer update them on subsequent tasks. In other words, their previous dual role as a weighting and buffer-storage mechanism now collapses to the former only. In Table 6, we illustrate the performance of CLOPS with and without the weighting of replayed instances using the task-instance parameters.

We find that weighting *replayed* instances with frozen task-instance parameters is a detriment to backward transfer. For example, in the Class-IL scenario, BWT = 0.053 and 0.042 for CLOPS without and with the weighting coefficient, respectively. This can be seen across the continual learning scenarios. We hypothesize that this behaviour is due to the 'down-weighting' of replayed instances. More specifically, since task-instance parameters, $\beta < 1$, networks end up learning less from replayed instances.

Supplementary Table 6. Performance of CL strategies in the three continual learning scenarios with and without weighted replayed instances. Storage and acquisition fractions are $b = 0.25$ and $a = 0.50$, respectively. Mean and standard deviation are shown across five seeds.

Method	Average AUC	BWT	BWT_t	BWT_λ
Class-IL				
CLOPS	0.796 ± 0.013	**0.053 ± 0.023**	**0.018 ± 0.010**	**0.008 ± 0.016**
CLOPS weighted	**0.800 ± 0.006**	0.042 ± 0.020	0.005 ± 0.014	-0.014 ± 0.016
Time-IL				
CLOPS	**0.834 ± 0.014**	**-0.018 ± 0.004**	**-0.007 ± 0.003**	0.007 ± 0.003
CLOPS weighted	0.818 ± 0.012	-0.024 ± 0.012	-0.011 ± 0.006	0.007 ± 0.002
Domain-IL				
CLOPS	0.731 ± 0.001	**-0.011 ± 0.002**	**-0.020 ± 0.004**	**-0.019 ± 0.009**
CLOPS weighted	**0.741 ± 0.007**	-0.016 ± 0.007	-0.024 ± 0.001	-0.024 ± 0.003

Effect of Number of Monte Carlo Samples, T

The functionality of uncertainty-based acquisition functions such as BALD_{MCD} is dependent upon a reasonable approximation of the region of uncertainty in the hypothesis space. Improved approximations are usually associated with an increased number of Monte Carlo samples. In all experiments so far, $T = 20$ MC samples were used. In this section, we repeat a subset of these experiments with $T = (5, 10, 50)$ and illustrate their results in Table 7.

As expected, there exists a proportional relationship between the number of MC samples, T, and the performance of the network in terms of average AUC and backward transfer. For instance, as $T = 5 \rightarrow 50$, the BWT $= 0.045 \rightarrow 0.061$. This observation implies that an improved approximation of the region of uncertainty can lead to the acquisition of instances from the buffer that increase the magnitude of *constructive* interference.

Supplementary Table 7. Performance of our strategy in the Class-IL scenario with different numbers of MC samples, T. Storage and acquisition fractions are $b = 0.25$ and $a = 0.50$, respectively. Results are shown across five seeds.

MC Samples T	Average AUC	BWT	BWT_t	BWT_λ
5	0.765 ± 0.013	0.045 ± 0.019	0.011 ± 0.016	0.002 ± 0.024
10	0.781 ± 0.016	0.051 ± 0.020	0.014 ± 0.018	0.002 ± 0.024
20	**0.796** ± **0.013**	0.053 ± 0.023	0.018 ± 0.010	0.008 ± 0.016
50	0.785 ± 0.023	**0.061** ± **0.009**	**0.023** ± **0.008**	**0.008** ± **0.012**

Effect of Type of Acquisition Function, α

Our modular buffer-acquisition strategy provides researchers with the flexibility to choose their acquisition function of interest. Beyond BALD_{MCD}, we repeat a subset of our experiments using two recently-introduced acquisition functions, BALD_{MCP} and BALC_{KLD}, which acquire instances based on how sensitive a network is to their perturbed counterpart[5]. We define these functions below and illustrate the results in Table 8.

Definitions of Acquisition Functions

$$\begin{aligned}\mathbf{BALD_{MCP}} &= \text{JSD}(p_1, p_2, \ldots, p_T) \\ &= \text{H}(p(y|x)) - \mathbb{E}_{p(z|D_{train})}\left[\text{H}(p(y|x,z))\right]\end{aligned} \tag{7}$$

$$\begin{aligned}\text{H}(p(y|x)) &= \text{H}\left(\int p(y|z)p(z|x)dz\right) \\ &= \text{H}\left(\int p(y|z)q_\phi(z|x)dz\right) \\ &\approx \text{H}\left(\frac{1}{T}\sum_{t=1}^{T} p(y|\hat{z}_t)\right)\end{aligned} \tag{8}$$

where z represents the perturbed input, T is the number of Monte Carlo samples, and $\hat{z}_t \sim q_\phi(z|x)$ is a sample from some perturbation generator.

$$\begin{aligned}\mathbb{E}_{p(z|D_{train})}\left[\text{H}(p(y|x,z))\right] &= \mathbb{E}_{q_\phi(z|x)}\left[\text{H}(p(y|x,z))\right] \\ &\approx \frac{1}{T}\sum_{t=1}^{T}\left[\text{H}(p(y|\hat{z}_t))\right] \\ &= \frac{1}{T}\sum_{t=1}^{T}\left[-\sum_{c=1}^{C} p(y=c|\hat{z}_t)\log p(y=c|\hat{z}_t)\right]\end{aligned} \tag{9}$$

$$\mathbf{BALC_{KLD}} = \mathscr{D}_{KL}(\mathscr{N}(\mu(x), \Sigma(x) \parallel \mathscr{N}(\mu(z), \Sigma(z))) \tag{10}$$

where $\mu = \frac{1}{T}\sum_{t=1}^{T} p(y|\hat{\omega}_t, x)$ is the empirical mean of the posterior distributions across T MC samples and $\hat{\omega} \sim q_\theta(\omega)$ represents parameters sampled from the MC distribution as in[6]. $\Sigma = (Y-\mu)^T(Y-\mu)$ is the empirical covariance matrix of the posterior distributions where $Y \in \mathbb{R}^{TxC}$ is the matrix of posterior distributions for all MC samples.

Results

We show that the type of acquisition function can have a large effect on the final performance of a CL algorithm and the degree of constructive interference that is experienced. Such a finding justifies our use of BALD_{MCD} for all experiments conducted in the main manuscript.

Supplementary Table 8. Performance of CLOPS ($b = 0.25$ and $a = 0.5$) in the Class-IL scenario with different acquisition functions. Mean and standard deviation values are shown across five seeds.

Acquisition Function	Average AUC	BWT	BWT_t	BWT_λ
BALD_{MCD}	**0.796 ± 0.013**	0.053 ± 0.023	**0.018 ± 0.010**	**0.008 ± 0.016**
BALD_{MCP}	0.674 ± 0.042	**0.068 ± 0.052**	(0.107 ± 0.035)	(0.091 ± 0.034)
BALC_{KLD}	0.716 ± 0.039	(0.036 ± 0.035)	(0.104 ± 0.039)	(0.104 ± 0.051)

Effect of Hyperparameters on MIR

In this section, we aim to provide a more holistic evaluation of our adaptation of MIR. To do so, we vary the two hyper-parameters involved in the implementation and observe their impact on the performance of the models in the Class-IL scenario. These hyper-parameters include the number of instances acquired from the buffer to perform the MIR search (Acquisition Fraction) and the ratio of replayed to current-task instances in each mini-batch (Ratio). In Fig. 10, we present the performance of MIR as a function of the acquisition fraction. In Fig. 9, we present the performance of MIR as a function of the ratio of replayed to current-task instances in each mini-batch.

Acquisition Fraction

Supplementary Table 9. Performance of MIR in the Class-IL scenario with different acquisition fractions. Larger acquisition fractions mean that a larger subset of the buffer is searched for maximally-interfered instances. The ratio of replayed to current-task instances is fixed at 1. Mean and standard deviation values are shown across five seeds.

Acquisition Fraction	Average AUC	BWT	BWT_t	BWT_λ
0.1	0.799 ± 0.006	0.046 ± 0.022	-0.005 ± 0.031	-0.026 ± 0.028
0.25	**0.807** ± **0.010**	0.043 ± 0.017	0.013 ± 0.016	-0.007 ± 0.022
0.5	0.753 ± 0.014	0.009 ± 0.018	0.001 ± 0.025	-0.046 ± 0.022
1	0.800 ± 0.013	**0.063** ± **0.023**	**0.039** ± **0.024**	**0.021** ± **0.023**

Ratio of Replayed to Current-task Instances

Supplementary Table 10. Performance of MIR in the Class-IL scenario with different ratios of replayed instances to current-task instances in the mini-batch. Larger ratios mean that greater emphasis is placed on replayed instances than on current-task instances. The acquisition fraction is fixed at 0.5. Mean and standard deviation values are shown across five seeds.

Ratio	Average AUC	BWT	BWT_t	BWT_λ
1:2	**0.801** ± **0.013**	**0.065** ± **0.025**	0.011 ± 0.043	-0.012 ± 0.030
1:1	0.753 ± 0.014	0.009 ± 0.018	0.001 ± 0.025	-0.046 ± 0.022
2:1	0.763 ± 0.012	0.016 ± 0.010	0.024 ± 0.017	-0.020 ± 0.021
4:1	0.752 ± 0.016	0.020 ± 0.021	0.030 ± 0.016	-0.013 ± 0.021

References

1. Hannun, A. Y. *et al.* Cardiologist-level arrhythmia detection and classification in ambulatory electrocardiograms using a deep neural network. *Nat. Medicine* **25**, 65 (2019).
2. Zheng, J. *et al.* A 12-lead electrocardiogram database for arrhythmia research covering more than 10,000 patients. *Sci. Data* **7**, 1–8 (2020).
3. Perez Alday, E. A. *et al.* Classification of 12-lead ECGs: the PhysioNet - computing in cardiology challenge 2020 (version 1.0.1). *PhysioNet* (2020).
4. Clifford, G. D. *et al.* Af classification from a short single lead ECG recording: the physionet/computing in cardiology challenge 2017. In *2017 Computing in Cardiology*, 1–4 (2017).
5. Kiyasseh, D., Zhu, T. & Clifton, D. A. Socal: Selective oracle questioning for consistency-based active learning of cardiac signals (2020). 2004.09557.
6. Gal, Y., Islam, R. & Ghahramani, Z. Deep Bayesian active learning with image data. In *Proceedings of the 34th International Conference on Machine Learning-Volume 70*, 1183–1192 (JMLR. org, 2017).
