# Supplementary material for: A clinical deep learning framework for continually learning from cardiac signals across diseases, time, modalities, and institutions
Source: Nat Commun. 2021 Jul 9;12:4221. doi: 10.1038/s41467-021-24483-0 (PMC8270996; doi:10.1038/s41467-021-24483-0)
Supplement: Supplementary file 3 — Reporting Summary [file 41467_2021_24483_MOESM3_ESM.pdf]

nature research

Corresponding author(s): Dani Kiyasseh

Last updated by author(s): 2021/06/05

Reporting Summary

Nature Research wishes to improve the reproducibility of the work that we publish. This form provides structure for consistency and transparency in reporting. For further information on Nature Research policies, see our Editorial Policies and the Editorial Policy Checklist.

Statistics

For all statistical analyses, confirm that the following items are present in the figure legend, table legend, main text, or Methods section.

n/a	Confirmed	
☐	☒	The exact sample size (*n*) for each experimental group/condition, given as a discrete number and unit of measurement
☐	☒	A statement on whether measurements were taken from distinct samples or whether the same sample was measured repeatedly
☒	☐	The statistical test(s) used AND whether they are one- or two-sided<br>*Only common tests should be described solely by name; describe more complex techniques in the Methods section.*
☐	☒	A description of all covariates tested
☒	☐	A description of any assumptions or corrections, such as tests of normality and adjustment for multiple comparisons
☐	☒	A full description of the statistical parameters including central tendency (e.g. means) or other basic estimates (e.g. regression coefficient) AND variation (e.g. standard deviation) or associated estimates of uncertainty (e.g. confidence intervals)
☒	☐	For null hypothesis testing, the test statistic (e.g. *F*, *t*, *r*) with confidence intervals, effect sizes, degrees of freedom and *P* value noted<br>*Give P values as exact values whenever suitable.*
☒	☐	For Bayesian analysis, information on the choice of priors and Markov chain Monte Carlo settings
☒	☐	For hierarchical and complex designs, identification of the appropriate level for tests and full reporting of outcomes
☒	☐	Estimates of effect sizes (e.g. Cohen's *d*, Pearson's *r*), indicating how they were calculated

Our web collection on statistics for biologists contains articles on many of the points above.

Software and code

Policy information about availability of computer code

Data collection	Since all data are publicly-available (see below), we did not perform any primary data collection.
Data analysis	https://github.com/danikiyasseh/CLOPS, Python 3, PyTorch 1.6.0

For manuscripts utilizing custom algorithms or software that are central to the research but not yet described in published literature, software must be made available to editors and reviewers. We strongly encourage code deposition in a community repository (e.g. GitHub). See the Nature Research guidelines for submitting code & software for further information.

Data

Policy information about availability of data

All manuscripts must include a data availability statement. This statement should provide the following information, where applicable:

- Accession codes, unique identifiers, or web links for publicly available datasets
- A list of figures that have associated raw data
- A description of any restrictions on data availability

All data are publicly-available at the following sites: https://irhythm.github.io/cardiol_test_set/ and https://figshare.com/collections/ChapmanECG/4560497/2 and https://physionetchallenges.org/2020/ and https://physionet.org/content/challenge-2017/1.0.0/

Field-specific reporting

Life sciences study design

All studies must disclose on these points even when the disclosure is negative.

Sample size	Cardiology dataset = 292 patients, Chapman dataset = 10,646 patients, PhysioNet 2020 dataset = 6,876 patients, PhysioNet 2017 dataset = 8,528 recordings. Details of the number of samples used during training, validation, and testing are provided in the attached supplementary files. Datasets were chosen to reflect the various continual learning scenarios (e.g., changes across disease, time, modalities, and institutions).
Data exclusions	We do not exclude any patients for training, validation, or testing.
Replication	Protocol is described in main manuscript and in-depth implementation details are provided in the attached supplementary files. All experiments were conducted at least five times (i.e., across five random seeds) and we report the mean and standard deviation across these five seeds.
Randomization	Experiments were conducted across five random seeds (i.e., random weight initializations and random data batches). Patients were split into training, validation, and test sets randomly based on a fixed seed.
Blinding	Blinding is not applicable in our case because we are not dealing with human subjects nor are we comparing two treatment paths. However, when comparing different experimental methods, we maintained the same experimental settings to allow for a fair comparison.

Reporting for specific materials, systems and methods

We require information from authors about some types of materials, experimental systems and methods used in many studies. Here, indicate whether each material, system or method listed is relevant to your study. If you are not sure if a list item applies to your research, read the appropriate section before selecting a response.

Materials & experimental systems

n/a	Involved in the study	
☒	☐	Antibodies
☒	☐	Eukaryotic cell lines
☒	☐	Palaeontology and archaeology
☒	☐	Animals and other organisms
☒	☐	Human research participants
☒	☐	Clinical data
☒	☐	Dual use research of concern

Methods

n/a	Involved in the study	
☒	☐	ChIP-seq
☒	☐	Flow cytometry
☒	☐	MRI-based neuroimaging
